# The dynamic reorganization of the default-mode network during a visual classification task

**DOI:** 10.3389/fnsys.2013.00034

**Published:** 2013-07-26

**Authors:** Wei Gao, John H. Gilmore, Sarael Alcauter, Weili Lin

**Affiliations:** ^1^Department of Radiology and Biomedical Research Imaging Center, University of North Carolina at Chapel HillChapel Hill, NC, USA; ^2^Department of Psychiatry, University of North Carolina at Chapel HillChapel Hill, NC, USA

**Keywords:** resting-state, functional connectivity, default-mode network, task-dependent, dynamic reorganization

## Abstract

The default-mode network has been reported to possess highly versatile and even contrasting functions but the underlying functioning mechanism remains elusive. In this study, we adopt a dynamic view of the default-mode network structure and hypothesize that it could potentially contribute to different functions through dynamic reorganization of its functional interaction pattern within and across network boundaries depending on the ongoing cognitive demands. With four experimental states and functional connectivity magnetic resonance imaging, we show that the default-mode network is characterized by within-network desynchronization and outside-network integration during the transition from resting state to an external visual classification task. Such default-mode network dynamics are task demand-dependent and return to their original status during the transition back to resting. More importantly, the degree of within-network desynchronization correlates with reaction time while the level of outside-network integration indexes task performance accuracy. Overall, the documented dynamic reorganization of the default-mode network and the significant behavioral correlations provide new insights into our understanding of this complex network and emphasize a dynamic view in future studies of its functioning mechanism.

## Introduction

One of the most important findings in recent neuroimaging research is the identification of the default-mode network (Shulman et al., 1997; Raichle et al., 2001), which encompasses a set of distributed brain regions including medial prefrontal cortex (MPFC), posterior cingulate cortex (PCC), bilateral lateral temporal cortex (LTC), and inferior parietal lobule (IPL) areas (Greicius et al., 2003; Buckner et al., 2008). Default-mode network regions are characterized by down-regulated activity during external goal-directed tasks when compared with unconstrained resting (Shulman et al., 1997; Raichle et al., 2001). The default-mode network represents one of the most robust findings and its structure has been consistently documented using both PET and functional connectivity MRI (fcMRI) (Biswal et al., 1995) in both human (Buckner et al., 2008) and animal models (Vincent et al., 2007). Unfortunately, despite of the high consistency in its structure, findings on its functional roles are much more divergent and a deterministic delineation remains an active area of research. Among the existing reports, one cohort provides evidence for its involvement in internal mentation such as episodic memory retrieval (Maguire, 2001; Svoboda et al., 2006), theory of mind (Amodio and Frith, 2006; Saxe and Powell, 2006), and envisioning the future (Schacter and Addis, 2007; Schacter et al., 2007), among others. In contrast, another group of studies provide supports for its active roles in allocating attentional resources to monitoring the external environment (Shulman et al., 1997; Gusnard and Raichle, 2001; Gilbert et al., 2006, 2007).

The mechanism of a single default-mode network supporting versatile functions is an active area of research (Andrews-Hanna et al., 2010; Kim, 2012; Mantini and Vanduffel, 2013). Among different approaches, recent studies looking at the task-dependent dynamic changes of this network represent a promising direction. For example, Frannsson (Fransson, 2006) has reported disrupted functional connectivity within certain default-mode network regions during an attention-demanding working memory task. Similarly, Hasson et al. (2009) also found dramatic decrease of default-mode network connectivity during language comprehension episodes. Such decrease of within-default-mode network connectivity during external goal-directed tasks is consistent with its role in task-independent internal mentation, which presumably would be suppressed during active performance of attention-demanding external tasks (Fransson, 2006). On the other hand, we and others have also consistently documented increased coupling between default-mode network regions and other task-related areas during a range of different tasks including natural movie watching (Gao and Lin, 2012), working memory (Bluhm et al., 2011), recollection (Fornito et al., 2012), and autobiographic planning (Spreng et al., 2010). Such increased coupling may reflect the default-mode network's active participation in the internal processing aspect of such tasks (Spreng et al., 2010; Fornito et al., 2012; Gao and Lin, 2012) or a general monitoring of the internal/external environment to facilitate task performance (Shulman et al., 1997; Gusnard and Raichle, 2001; Gilbert et al., 2006, 2007). Overall, previous findings suggest that an alternative and promising way to look at default-mode network's versatile functional roles is to characterize its dynamic reorganization across different brain states.

However, the dynamic reorganization of the default-mode network, including both task-dependent decrease and increase of functional synchronization, has rarely been systematically characterized based on the same task in a single study. Moreover, the behavioral significance of such two-way reorganization also remains to be determined. To this end, a four-stage experiment was designed in this study to specifically characterize the task-dependent, two-way reorganization of the default-mode network and its corresponding behavioral significance. Specifically, we tested the hypothesis that during the transition from unconstrained resting to external attention demanding tasks, the default-mode network would experience within-network desynchronization and outside-network integration to cope with the brain state shift. However, the opposite pattern should occur during the transition back from external tasks to rest. Moreover, if such task-dependent reorganization of the default-mode network represents true underlying functional reallocation, we would expect significant behavioral correlations. Specifically, the four stage experiment consisted of a pre-task resting state (R1), followed by two task states with lower (T1) and higher (T2) attentional demands, respectively, and finally a post-task resting state (R2). Both seed-based analysis and data driven independent component analysis (ICA) were conducted to show the dynamic changes of the default-mode network connectivity. Quantitative brain-behavior correlation analysis was finally carried out to examine the behavioral significance of these changes. Our experimental results confirmed all of our hypotheses and shed new light on a dynamic view of the default-mode network's functional organization.

## Materials and methods

### Subjects and experimental design

**Figure 1:**
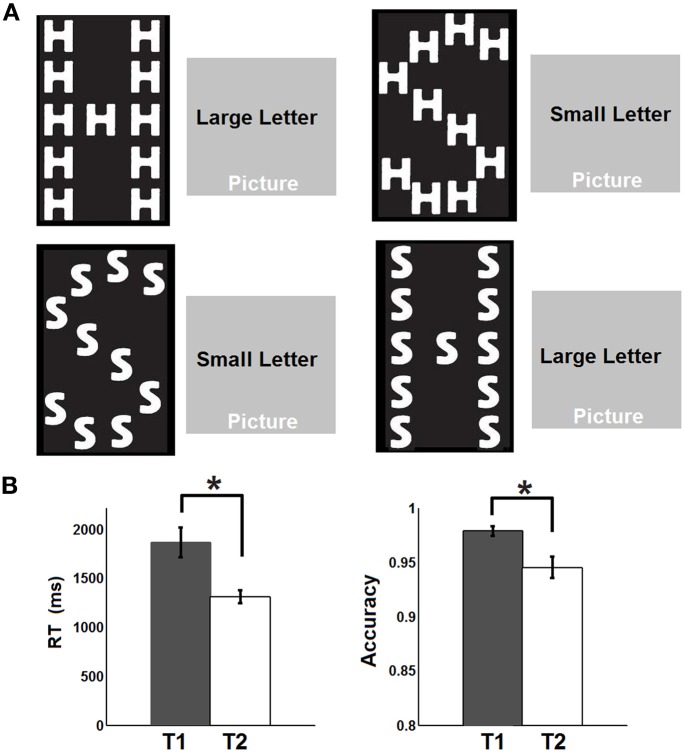
**Experimental design and behavioral performances**. **(A)** Experimental design: The subjects were asked to identify either the large, global letter or the small, local letter of a hierarchically organized object and respond by pressing the corresponding button on a serial response box. The cues to identify either the large or the small letter were overlapped on scenic pictures. **(B)** Response time (RT) and accuracy recorded during the two task states. Whiskers represent standard error of the mean. Asterisks represent statistically significant changes (FDR corrected *p* < 0.05) based on paired *t*-test between the two states.

A total of 19 healthy adult subjects (age 27–40, 5F, all right-handed) were recruited in this study. Informed consent was obtained from each participant and the experimental protocols were approved by the institutional review board. Each participant underwent four steady state fcMRI runs including a pre-task resting run (R1), a relaxed attentional task run (T1), an intense attentional task run (T2), and finally a post-task resting run (R2). For both the pre- and post-task resting runs (T1/T2), participants were asked to keep still in the scanner with their eyes closed. The task for the steady state task runs was similar to a previous study (Weissman et al., 2006) where the subjects were asked to classify the identity of either a large, global letter or small, local letters of a hierarchically organized object (Figure 1A) and respond by pressing the corresponding button on a serial response box using the thumb of each hand (i.e., left thumb for letter “H” and right thumb for letter “S,” respectively). The choice of this visual attention task stems from its effectiveness in perturbing default-mode network activity as reported in a previous study (Weissman et al., 2006). To introduce differential attentional demands, the participants were asked to perform the task at a relaxed pace during T1 but try their best to respond as fast as possible during T2. Throughout T1 and T2, all subjects performed the task in a continued manner (i.e., no blocks and no fixation) so that the next trial appeared immediately after the subject made a response to the previous trial. Accuracy was equally emphasized during both task runs. The cues to identify either the large or the small letters were overlapped on scenic pictures (Figure 1A) with the intention to increase the complexity of the task and engage more cognitive control functions. The target object, the cue and the scenic picture were randomly combined for each trial. There were both congruent and incongruent trials depending on whether the small and the big letters were the same or not (Figure 1A). The task was designed and presented using the Eprime software which also recorded the online response time (RT) and accuracy during both task runs. After the R1 run, all participants completed T1, T2 (order counterbalanced across subjects), followed by R2. Each fMRI run lasted 5 min during which 150 volumes were acquired.

### MR acquisition

All images were acquired using a Siemens Trio 3T MR scanner (Siemens Medical Inc., Erlangen, Germany). Before the fcMRI runs, anatomical images were acquired using a 3D MP-RAGE sequence and these images were subsequently used for spatial normalization. The imaging parameters were: repetition time (*TR*) = 1820 ms; echo time (*TE*) = 4.38 ms; inversion time = 1100 ms; 144 slices; and voxel size = 1 × 1 × 1 mm^3^. For the fcMRI scan, a T2^*^-weighted echo-planar imaging (EPI) sequence was used with the following imaging parameters: *TR* = 2000 ms, *TE* = 32 ms; 33 slices; and voxel size = 4 × 4 × 4 mm^3^. This sequence was repeated 150 times (5 min) for each experimental condition.

### Preprocessing

All Functional images were preprocessed using SPM8 software. The first 10 time points of the fcMRI data were excluded to allow magnetization to reach an equilibrium condition. Subsequently, images were corrected for slice timing, realigned to the second available scan in each functional series, registered to the Montreal Neurological Institute (MNI) template space and re-sliced to 3 mm cubic voxels. All images were then spatially smoothed with an 8 mm FWHM Gaussian kernel and band passed between 0.008 and 0.08 Hz. Regression analyses were further performed to remove nuisance signals from white matter, cerebral spinal fluid (CSF), global signal and six movement parameters. Given the recent report that subject motion alters the time courses of resting state functional data, introducing artificial correlation structures, even after spatial registration and regression of motion estimates (Power et al., 2012), we implemented a new method to control the frame-wise global signal change and displacement (threshold was chosen to be 0.5% BOLD signal and 0.5 mm, respectively (Power et al., 2012)). Briefly, if both measures of any volume of the functional series reached their respective thresholds, that volume, the one previous and the two after were removed. For R1, T1, and T2 run, all subjects demonstrated minimal motion artifacts and no volumes for any subject were removed. During the R2 run, three subjects demonstrated severe motion artifact and more than 60 volumes were removed based on the described motion correction procedure. Therefore, the data from these three subjects during R2 run were excluded from subsequent analysis. Nevertheless, the data of these three subjects during other runs (R1, T1, T2) were included. The usage of these data will be made clear in the corresponding text.

### Functional connectivity analysis

After preprocessing, the subsequent analyses were carried out using custom Matlab code and GIFT software (http://icatb.sourceforge.net/) (Calhoun et al., 2001). Seed-based analyses were firstly carried out to delineate the functional connectivity map of the default-mode network across the four experimental conditions (R1, T1, T2, and R2). Specifically, a spherical ROI (with a radius of 8 mm) was selected in the PCC (MNI: 0, −53, 26) (Andrews-Hanna et al., 2007; Van Dijk et al., 2010) as the seed region based on which a whole brain correlation analysis was carried out for each subject. After Fisher-Z transform, the first-level correlation maps were entered into a second-level random effect model and significant correlations were defined using two-way *t*-test at a FDR (Benjamini and Yekutieli, 2001) corrected level of *p* < 0.05. Note in this section of analyses, due to the exclusion of three subjects from R2 run, only the remaining 16 subjects with all four run data were included.

A preliminary check of the resulting four-state functional connectivity maps revealed that part of the regions appearing significantly connected to the default-mode network during R1 disappeared during T1/T2 and reappeared during R2 while another set of regions were absent from R1 map but appeared within T1/T2 maps and disappeared again in R2 map. Such dynamic changes are consistent with our within-network disruption and outside network integration hypothesis if we assume that the topology during resting states (R1/R2) represents the “true” default-mode network structure while that during tasks (T1/T2) represents task-dependent, “transient” coupling. To quantitatively examine such task-dependent changes of default-mode network connectivity, we opted to first define a mask as the union of the four significant connectivity maps (i.e., voxels that appear significant within at least one of the four connectivity maps were included) and search for statistically significant changes within this mask. The use of such a mask ensured that the detected voxels showing significant decreases in connectivity from resting to task states were within the original resting default-mode network while the detected voxels showing significant increase in connectivity were also significantly coupled with the default-mode network during task states (i.e., they not only showed relative changes but also were significantly coupled with the default-mode network during either resting or task brain state). Specifically, one-way repeated measure ANOVA was conducted on voxels within this mask based on the individual default-mode network connectivity values across the four stages to detect statistically significant changes (FDR corrected *p* < 0.05). A cluster size >5 was employed in the detection to minimize random noise. Subsequently, a retrospective paired *t*-test was conducted to classify the detected voxels into “increasing” and “decreasing” regions depending on whether they showed significant increase or decrease of connectivity during task states (mean of T1, T2) compared with the resting states (mean of R1, R2). Note we had performed a direct comparison between the R1 and R2 (paired *t*-test) states and no statistically significant changes in connectivity strengths were detected so the mean of R1 and R2 was used here to define task-dependent functional connectivity changes. As expected, all voxels detected to show significant changes in the repeated measures ANOVA step showed significant changes in the subsequent paired *t*-test hence were labeled as “decreasing” and “increasing” regions, respectively. Besides statistically changing voxels, the voxels that (1) showed no changes in connectivity based on the ANOVA step and (2) consistently appeared in the significant default-mode network connectivity maps across the four stages were also extracted and defined as “stable” regions.

To confirm that such increasing/decreasing connectivity patterns were not a phenomenon tied to the seed region PCC but rather represented a network-level behavior, functional ROIs were defined for the statistically increasing/decreasing regions as well as stable regions. Subsequently, connectivity changing patterns between all other “stable” default regions (besides PCC) and the detected changing regions (i.e., both increasing and decreasing regions) were also calculated and compared using repeated measure ANOVA. After that, a paired *t*-test was again performed between each set of two consecutive stages (i.e., between R1 and T1, between T1 and T2, and between T2 and R2) to show the detailed dynamic pattern of each pair-wise connection. After that, all pair-wise connections between stable regions and increasing/decreasing regions were separately averaged within individual subjects as measures of system-level connections (i.e., stable-increasing regions/stable-decreasing regions), which were then similarly compared across different experimental states to delineate the system-level connectivity changing patterns. Finally, connection within each system (i.e., stable, increasing, and decreasing regions) were averaged and similarly tested. Significance was defined at a FDR corrected level of *p* < 0.05.

To validate the findings from these seed based analyses, a data-driven method-group ICA was carried out using GIFT software (http://icatb.sourceforge.net/) (Calhoun et al., 2001). Specifically, the infomax algorithm (Bell and Sejnowski, 1995), which maximizes the information transfer of a network using non-linear functions, was applied for ICA analysis on dimension-reduced and concatenated data set to obtain a set of aggregate independent components for each experimental state. The number of components was determined using the minimum description length criteria (Li et al., 2007), which was 21, 21, 24 and 22 for R1, T1, T2 and R2, respectively. After group ICA, an automated template matching approach (Greicius et al., 2004) was employed to select the component comprising brain regions that best matched with the commonly observed brain regions in the default-mode network, including bilateral medial superior frontal and bilateral posterior cingulate gyrus (Raichle et al., 2001; Buckner et al., 2008) as defined in the AAL atlas (Tzourio-Mazoyer et al., 2002). This approach was applied to each of the four experimental states, respectively, and the best matching component was selected to represent the default-mode network for each state examined. To further test whether similar quantitative task-dependent changes could be detected using ICA-derived component scores, individual default-mode network components were back-reconstructed using GIFT software and entered into identical statistical analysis (repeated ANOVA followed by paired *t*-test) as described above to detect task-dependent changes in a data-driven fashion.

### Connectivity-behavior analysis

As the last step, connectivity-behavioral analyses were conducted to explore the behavioral significance of the detected functional connectivity changes. To take advantage of all the subjects in this correlation analysis, we included all 19 subjects based on their R1, T1, and T2 data (the three excluded datasets were from the R2 run). Specifically, the system-level changes of functional connectivity (FC) between the stable and the increasing/decreasing regions from resting (R1) to external tasks (T1/T2) were calculated [dFC (stable, decreasing/increasing)] and correlated with two task performance measures – accuracy and mean RT, to detect significant behavioral effect of the dynamic connectivity changes during both T1 and T2. Similar correlation procedures were also conducted for the mean connection strength within the three sub-systems. The significance of brain-behavior relationship was also defined as FDR corrected *p* < 0.05. To validate our findings, bootstrapping of the observed correlations was performed based on 1000 times resampling with replacement and the 95% bootstrap confidence interval was calculated for each correlation.

## Results

**Figure 2:**
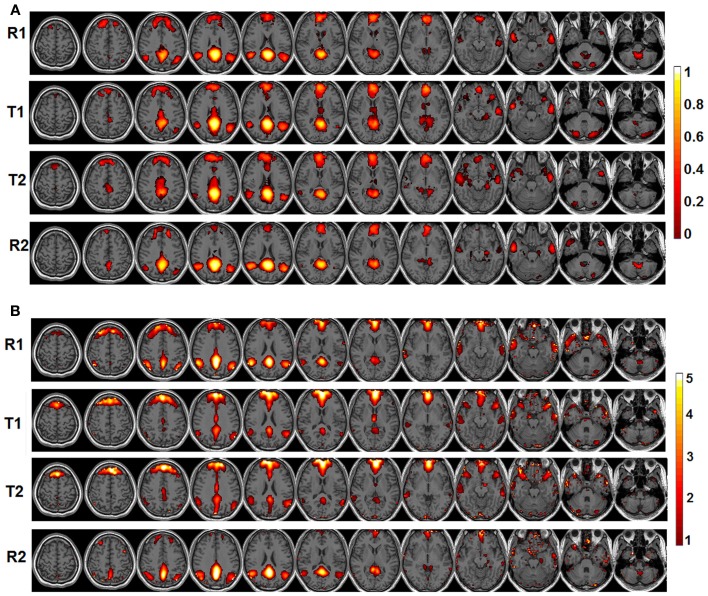
**The significant functional connectivity maps (*p* < 0.05, FDR corrected) of the default-mode network during four experimental states (pre-task resting state-R1; lower level external task state-T1; higher level external task state-T2; and post-task resting state-R2)**. **(A)** Results from seed-based functional connectivity analysis; color bar represents functional connectivity values; **(B)** Results from independent component analysis (ICA); color bar represents z-scores.

The behavioral data are presented in Figure 1B. Briefly, the RT is significantly faster during T2 than T1 [T1: 1864 ms (mean) ±152.8 ms (standard error of the mean); T2: 1310 ± 65.6 ms; *p* = 0.0018] while the accuracy is significantly lower during T2 than T1 (T1: 97.8 ± 0.46%; T2: 94.5 ± 0.99%; *p* = 0.0248). The significant default-mode network connectivity maps (FDR corrected *p* < 0.05) from both seed-based analysis and ICA are presented in Figure 2. For seed-based results (Figure 2A), despite the relatively stable default-mode network structure within its main components, dynamic changes do occur across different states. For example, the bilateral angular gyrus (ANG) and precuneus areas showing significant connectivity during both R1 and R2 dramatically shrink during both T1 and T2. In contrast, the anterior/middle cingulate areas, not typically involved in the default-mode network (not present during either R1 or R2), become significant during T1 and T2. As mentioned above, if we assume that the connectivity map during resting states represents the “true” default-mode network topology while that during task states stands for task-dependent, “transient” coupling, these dynamic patterns agree well with our within-network desynchronization and outside-network integration hypotheses. As expected, the default-mode network's topologies from ICA analysis (Figure 2B) are generally consistent with those from seed-based analysis (spatial correlation values between corresponding pairs of maps from two methods during R1, T1, T2, and R2 are 0.71, 0.54, 0.50, 0.68, respectively, with *p*-values less than 1e-6 for all correlations). More importantly, the relative task-dependent changes in topology seem also to be largely consistent between the results from the two independent approaches. However, there are also obvious differences. For example, the posterior cingulate/precuneus areas during T1/T2 are relatively more spatially restricted while the medial prefrontal areas are more emphasized in Figure 2B than in Figure 2A. Such differences may potentially reflect the methodological differences underling the calculation of these maps: seed based correlation analysis assesses the bivariate relationship between target and seed regions while ICA maximizes within-map correlation with the between-map spatial independence constraint in a multivariate fashion.

**Figure 3:**
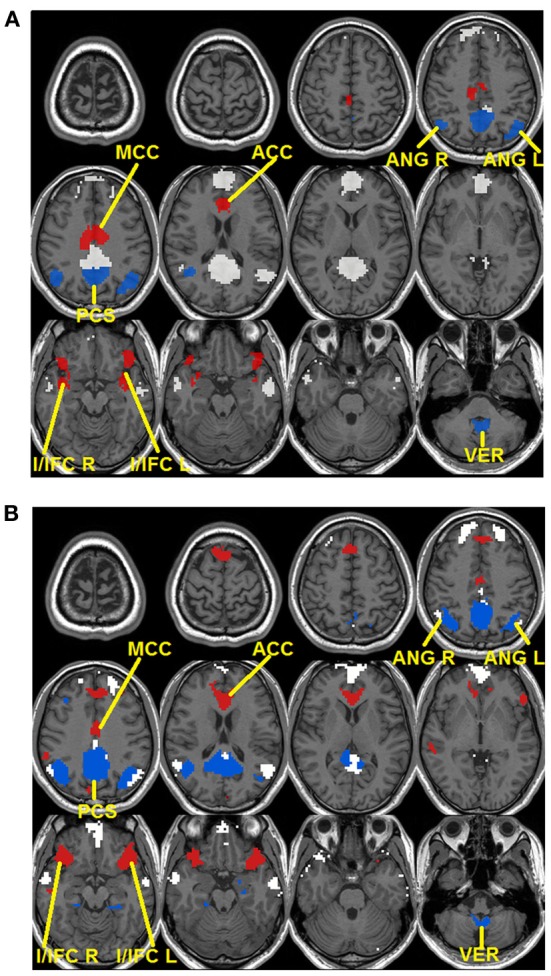
**Dynamic reorganization of the default-mode network across different experimental states**. Results from both seed-based functional connectivity analysis **(A)** and independent component analysis (ICA) **(B)** are presented. The regions demonstrating statistically significant decrease in connectivity during tasks (T1 and T2) compared with resting state (R1 and R2) at FDR corrected *p* < 0.05 are shown in blue colors while the regions showing the opposite pattern [i.e., statistically significant increase in connectivity during tasks (T1 and T2) compared with resting state (R1 and R2)] are shown in red. Besides, stable regions that remain unchanged and within the significant maps across the four states are shown in white color for comparison.

To validate these observations, quantitative detection results of significantly changing regions are presented in Figure 3. For seed-based analysis results (Figure 3A), areas within the precuneus (PCS), bilateral ANG, and vermis area (VER) demonstrate a reduction in connectivity strengths (FDR corrected *p* < 0.05, blue color, 4 regions, Figure 3A) from resting (R1 and R2) to external tasks (T1 and T2). These regions generally agree with those areas that disappear from the default-mode network maps during the transition from resting to tasks as shown in Figure 2A, which is highly consistent with our within-network desynchronization hypothesis. In contrast, bilateral insula/inferior frontal cortex (I/IFC), anterior cingulate cortex (ACC), and middle cingulate cortex (MCC) demonstrate increased connectivity from resting to external tasks (FDR corrected *p* < 0.05, red color, 4 regions, Figure 3A). Again, these regions show high consistency with those that emerge within the default maps during the transition from resting to external tasks (Figure 2A), which is in line with our outside-network integration expectation. Finally, the stable regions showing no statistically significant changes in connectivity and present within the significant functional connectivity maps of all four experimental stages are also presented (including part of PCC, MPFC, bilateral IPL, and bilateral LTC, white color, Figure 3A) for comparison. Again, the quantitatively defined changing regions from ICA analysis (Figure 3B) are highly consistent with those in Figure 3A. Such converging results strongly support the robustness of the dynamic reorganization pattern of the default-mode network across different experimental conditions. Given the similarity, the following analyses were carried out based on regions defined from seed-based results (Figure 3A).

**Figure 4:**
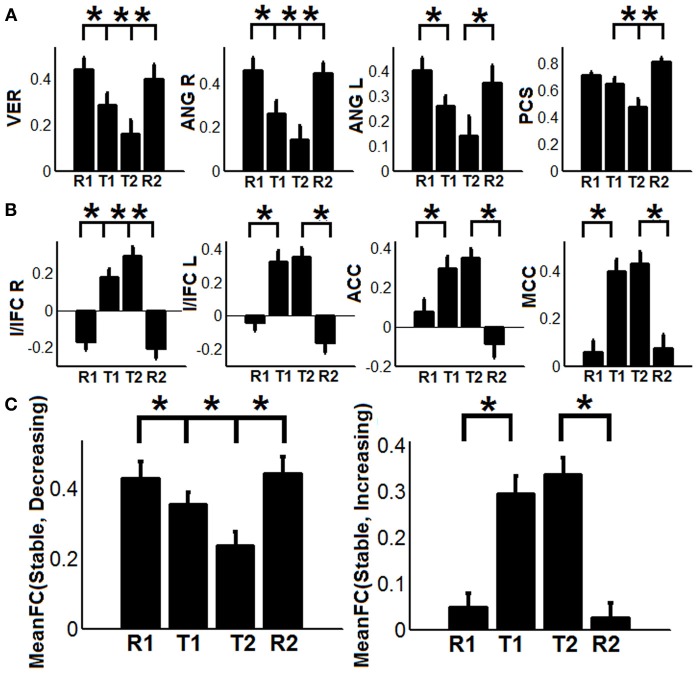
**The dynamic changes of connectivity among stable regions, increasing regions, and decreasing regions**. **(A)** Functional connectivity changes between the posterior cingulate (PCC) region and decreasing regions (PCS: precuneus, ANG: angular gyrus, VER: vermis area, blue regions in Figure 3A); **(B)** Functional connectivity changes between the PCC region and increasing regions (I/IFC, insula/inferior frontal cortex; ACC, anterior cingulate cortex; and MCC, middle cingulate cortex, red regions in Figure 3A); **(C)** The dynamic changes of system-level mean functional connectivity (FC) between stable regions and decreasing regions [MeanFC (Stable, Decreasing), left column] and increasing regions [MeanFC (Stable, Increasing), right column]. Asterisks represent statistically significant changes between two consecutive brain states (based on paired *t*-test thresholded at FDR corrected *p* < 0.05). All whiskers in the figure represent standard error of the mean.

**Figure 5:**
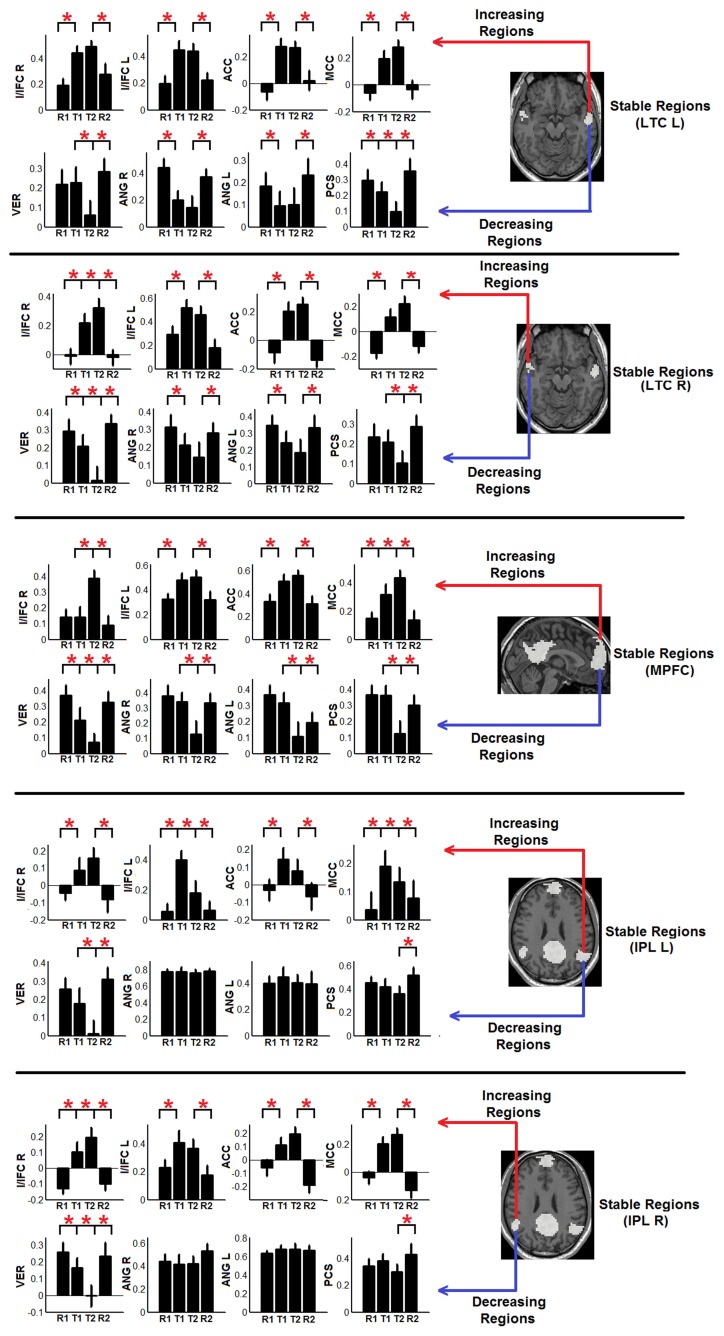
**The dynamic changes of individual connectivity patterns between the other five stable regions besides PCC region (i.e., bilateral LTC, lateral temporal cortex; MPFC, medial prefrontal cortex; and bilateral IPL, inferior parietal lobule; white regions in Figure 3A) and the detected decreasing/increasing regions (Decreasing regions—PCS, precuneus; ANG, angular gyrus; VER, vermis area; blue regions in Figure 3A; increasing regions—I/IFC, insula/inferior frontal cortex; ACC, anterior cingulate cortex; and MCC, middle cingulate cortex; red regions in Figure 3A)**. Each panel shows the connectivity changing pattern between one stable region (shown in the brain map in the right part of the panel) and the two set of increasing (upper row in each panel) and decreasing regions (bottom row in each panel). Red asterisks represent statistically significant changes between two consecutive brain states (based on paired *t*-test thresholded at FDR corrected *p* < 0.05). All whiskers in the figure represent standard error of the mean.

**Figure 6:**
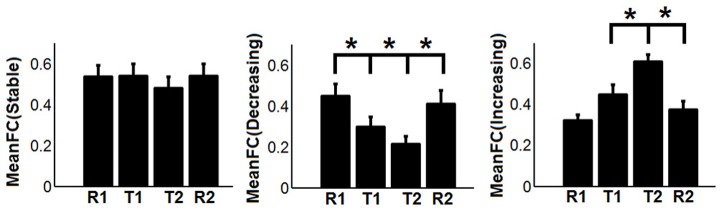
**The dynamic changes of mean functional connectivity (FC) within the three systems of regions-the stable regions [MeanFC (Stable): mean functional connectivity among stable regions], the decreasing regions [MeanFC (Decreasing): mean functional connectivity among decreasing regions], and the increasing regions [MeanFC (Increasing): mean functional connectivity among increasing regions]**. Asterisks represent statistically significant changes between two consecutive brain states (based on paired *t*-test thresholded at FDR corrected *p* < 0.05). Whiskers in the figure represent standard error of the mean.

To show the detailed dynamic functional connectivity patterns among the three systems of regions across the four experimental states, the state-specific connectivity strength between PCC and the two categories of changing regions and the corresponding statistical comparison results are shown in Figures 4A,B. Note all pair-wise connections show significant differences across the four experimental states based on repeated measure ANOVA testing (FDR corrected *p* < 0.05). *Post-hoc* paired *t*-tests reveal that the functional connectivity between PCC and most decreasing regions is significantly reduced from R1 to T1 (except for PCS), further diminishes from T1 to T2 (except for ANG L), and increases from T2 to R2, returning to the R1 level. For the increasing regions, all pair-wise connections start from very low or even negative but significantly increase to positive from R1 to T1, show moderate enhancement from T1 to T2 (only I/IFC R reaches significance), and significantly decrease back from T2 to R2. To demonstrate that such increasing/decreasing connectivity pattern is not a phenomenon tied to the seed region PCC but rather represents a network-level behavior, the system-level mean connectivity change patterns are shown in Figure 4C. As expected, the interaction between stable regions and decreasing regions shows a significant decrease from R1 to T1 (R1: 0.43 ± 0.047; T1: 0.35 ± 0.034; *p* = 0.02), a further decrease from T1 to T2 (T2: 0.24 ± 0.041; *p* = 0.0058), and finally a significant increase (R2: 0.45 ± 0.042; *p* = 0.0026) from T2 to R2. In contrast, the interaction between stable and increasing regions shows a significant increase from R1 to T1 (R1: 0.05 ± 0.028; T1: 0.30 ± 0.037; *p* < 0.001), a further non-significant increase from T1 to T2 (T2: 0.34 ± 0.035; *p* > 0.05), and a significant reduction from T2 to R2 (R2: 0.03 ± 0.028; *p* < 0.001). Additionally, to examine the potential region-specific dynamic patterns, the state-dependent connection strength between each of the five remaining stable regions and the increasing/decreasing regions are shown in Figure 5. Highly consistent task-dependent changing patterns are observed with the exception that the bilateral IPL regions seem to show less dynamic changes with the spatially adjacent bilateral ANG/PCS areas (Figure 5). Finally, to examine the within-system connectivity changes and compare with between-system dynamics, the state-dependent mean connectivity values within each category of regions are shown in Figure 6. As shown, the stable regions maintain a consistently high level of connectivity throughout the four experimental states while the decreasing/increasing regions show task-dependent desynchronization/enhancement pattern similar to their interaction with the stable regions. Note when we compared all the above mentioned measures between the two resting states (R1 and R2), no statistical significance was detected.

**Figure 7:**
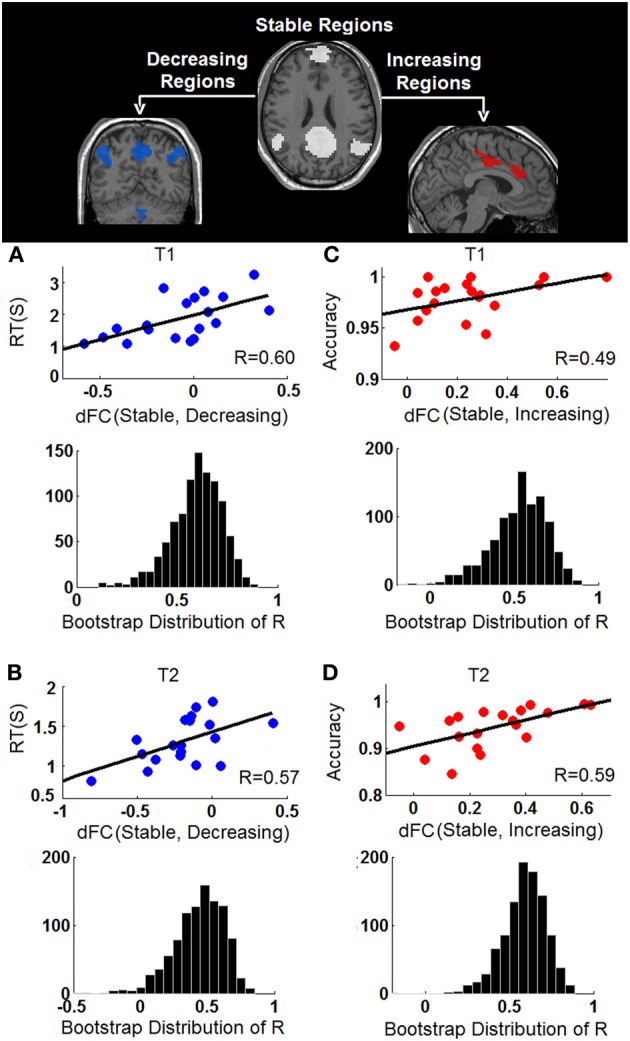
**Behavioral correlations of the task-dependent changes of functional connectivity**. **(A)** The correlation between changes of stable-decreasing regions connectivity [dFC (Stable, Decreasing)] and response time (RT) during T1; **(B)** The correlation between changes of stable-decreasing regions connectivity [dFC (Stable, Decreasing)] and response time (RT) during T2; **(C)** The correlation between changes of stable-increasing regions connectivity [dFC (Stable, Increasing)] and accuracy during T1; **(D)** The correlation between changes of stable-increasing regions connectivity [dFC (Stable, Increasing)] and accuracy during T2. In each panel, the observed correlation is shown in upper row and the histogram of the corresponding bootstrapped correlation values (*n* = 1000) is presented in the bottom row.

The behavioral significance of the detected task-dependent default-mode network reorganization was examined and is shown in Figure 7. Our system-level results show significant positive correlations between connectivity changes of dFC (stable, decreasing) and RT during both T1 (*R*^2^/*R* = 0.3622/0.6018, *p* = 0.0064, 95% bootstrap confidence interval for R: [0.2865, 0.7927], Figure 7A), and T2 (*R*^2^/*R* = 0.3301/0.5745, *p* = 0.01, 95% bootstrap confidence interval for R: [0.1560, 0.7973], Figure 7B), indicating that the more significant the within-network desynchronization (w.r.t the decreasing regions), the faster the response is. On the other hand, significant positive correlations are observed between dFC (stable, increasing) and accuracy during T1 (*R*^2^/*R* = 0.2400/0.4899, *p* = 0.03, 95% bootstrap confidence interval for R: [0.0703, 0.8528], Figure 7C), and T2 (*R*^2^/*R* = 0.3453/0.5876, *p* = 0.0082, 95% bootstrap confidence interval for R: [0.2748, 0.7905], Figure 7D), suggesting the more enhancement in outside-network integration (w.r.t the increasing regions), the more accurate the response would be. No correlation is observed between dFC (stable, decreasing) and accuracy or between dFC (stable, increasing) and RT, indicating a potential disassociation effect on such brain-behavioral relationships. Moreover, no significant correlation is observed between within-system connectivity changes (Figure 6) and behavioral measures.

## Discussion

The default-mode network is a functionally versatile network attracting considerable interests in studies of development (Fair et al., 2008; Gao et al., 2009, 2012), aging (Damoiseaux et al., 2008), and diseases (Greicius et al., 2004; Whitfield-Gabrieli et al., 2009), yet how its potential functions in contrasting domains are fulfilled remains an active area of research. In this study, we hypothesize a dynamic reorganization model of the default-mode network in support of its versatile functions. As expected, we detect a set of “decreasing” regions that are within the default-mode network during resting state but become increasingly desynchronized as external attention demands increase. The degree of this within-network desynchronization significantly correlates with individual RT. In contrast, we also find another set of “increasing” regions that do not typically belong to the resting state default-mode network but become increasingly integrated with it when external attentional demands increase. Behaviorally, the degree of this outside-network integration significantly indexes individual variability in task performance accuracy.

### The within-network desynchronization from resting to external attention tasks

The default-mode network is typically defined as a coherent system during resting state (Shulman et al., 1997; Raichle et al., 2001; Greicius et al., 2003; Buckner et al., 2008). However, evidence exists supporting the heterogeneity of this network (Buckner et al., 2008; Andrews-Hanna, 2012). In this study, we show that a subset of typical default-mode network regions becomes increasingly desynchronized when external attention demands increase. Specifically, these regions include precuneus (PCS), bilateral ANG, and VER in the cerebellum (Figure 3). Consistent with our findings, Fransson (Fransson, 2006) also reported a quantitative reduction of functional connectivity between PCC and several regions of the default-mode network during a working memory task, including PCS, and bilateral ANG, among others, which largely agree with the decreasing regions detected in this study. The precuneus, situated posterior to the branch of the cingulate sulcus and anterior to the triangular-shaped convolution of the cuneus, has been frequently associated with a wide spectrum of highly integrated operations, especially those that are self-related such as self-centered imagery, episodic memory retrieval and experience of agency (i.e., the feeling of being causally involved in an action). For a review on the functions related to PCS, see Cavanna and Trimble (Cavanna and Trimble, 2006). Consistently, located in the occipital-parietal junction areas where both visual and somatosensory signals are processed, the ANG is assumed to be involved in the elaboration of ones' own body image which also critically contributes to the sense of agency (Farrer and Frith, 2002; Farrer et al., 2003). For example, Farrer and Frith (2002) reported that the ANG was activated when people made the correct judgment that it was not themselves but other persons that caused the action visualized on the screen. Taken together, both the precuneus and ANG areas, detected to be less integrated with the other parts of the default-mode network during external tasks in this study (Figures 2, 3), seem to be involved in self-related cognitive judgment, especially the sense of agency (Farrer and Frith, 2002; Farrer et al., 2003; Cavanna and Trimble, 2006). Therefore, it is likely that when most of the brain's cognitive processing resources are shifted to external tasks (i.e., from resting R1 to tasks T1/T2), such internal-based, task-independent cognition is largely suppressed hence the high level of synchronization between these regions and the other part of the default-mode network is no longer maintained. Interestingly, the cerebellum VER is also among such regions. Although the cerebellum has been typically linked with motor coordination and control, its involvement in higher-order cognition has been increasingly recognized (Schmahmann and Caplan, 2006; Buckner et al., 2011). Particularly, the VER has been reported to contain axonal dopamine transporter immunoreactivity and is related to incentive-related behaviors (Anderson et al., 2006) and emotional processing (Lane et al., 1997b; Reiman et al., 1997). It is plausible that such reward/emotion processing is also suppressed during the external visual classification tasks as adopted in this study leading to the observed functional connectivity disruption. Finally, the within-system connections among these decreasing regions also show task-dependent disruption (Figure 6), reinforcing this suppression hypothesis.

However, the MPFC, PCC, bilateral IPL, and LTC areas remain highly synchronized across different cognitive states (Figures 2, 3). One possible explanation is that such persistent connectivity within core default-mode network regions may represent resources pre-budgeted for subconscious processes that do not directly compete with the ongoing cognitive operations. Results from both an anesthetized monkey study (Vincent et al., 2007) and a human study (Greicius et al., 2008) showing persistent default-mode network connectivity among highly consistent regions during anesthesia-induced unconscious states support this postulation. Specifically, such subconscious processes may include the maintenance of self-representation as previously suggested by Fransson (2006). Conceptually, we could not lose track of ourselves even when most of our conscious processing resources are directed to external cues (Fransson, 2006). However, further studies are needed to conclusively delineate the exact functional role of such tight and stable functional coordination among this cohort of core default-mode network regions across different brain states.

### The outside-network integration from resting to external attention tasks

In contrast to the regions showing disrupted connectivity with the default-mode network during the transition from resting to external attention tasks, there is another set of regions, typically outside the default-mode network, that shows enhanced integration and becomes significantly synchronized with the default-mode network (Figures 2, 3). These regions include bilateral insula/inferior frontal cortex (I/IFG), anterior cingulate cortex (ACC), and middle cingulate cortex (MCC). The insula, frequently activated in a series of goal-directed tasks (Duncan and Owen, 2000), is a functionally heterogeneous structure with versatile functions including interoceptive awareness, emotional responses, attentional budgeting, and high-level cognitive control (Sridharan et al., 2008; Menon and Uddin, 2010; Chang et al., 2012). Moreover, the anterior insula has also been associated with error perception (Ullsperger et al., 2010). The anterior cingulate is mostly well-known for its role in conflict monitoring, error detection/processing, and attention allocation in goal-directed tasks (Carter et al., 1999; Braver et al., 2001). Finally, the middle cingulate cortex, although frequently reported to be related to mentalization (Lombardo et al., 2010), has also been associated with inhibitory control and working memory functions (Kana et al., 2007). Interestingly, the insula and ACC regions have been previously characterized to together form a unified “salience network” whose primary function is to “integrate highly processed sensory data with visceral, autonomic, and hedonic “markers” so that the organism can decide what to do (or not to do) next” (Seeley et al., 2007). In this study, the task design involves both congruent and incongruent trials; the subjects have to inhibit the distraction of the pictures to read the cues and volitionally set the pace for the whole task performance. Therefore, it is not difficult to imagine the critical role of the identified salience network regions in integrating multi-domain information, processing conflicts, and identifying the most salient signals (from both the internal and external environment) during this task (Seeley et al., 2007). In this regard, the increased coupling between the stable default-mode network regions and salience network regions is plausible. First, the default-mode network regions may participate in assessing internal information to help gauge the salience of such stimuli. Potential internally salient events (e.g., pain, hunger) may need urgent attention despite the focus on external tasks and increased coordination among involved parties is preferred to promptly process such events, if they occurred, given the limited resources during such external task performance. Consistent with this hypothesis, the medial prefrontal area of the default-mode network is frequently associated with processing of various types of internal information including autonomic control (Critchley et al., 2000), emotional introspection (Lane et al., 1997a), and a “default” state of semantic processing (Binder et al., 1999; Gusnard et al., 2001; Raichle et al., 2001). On the other hand, given previous reports of the default-mode network's active role in monitoring the external world (Gilbert et al., 2006, 2007; Hahn et al., 2007), the default-mode network may also increase coupling with salience network regions to help assess the salience of a variety of different external stimuli to correctly perform the required tasks. However, these two possibilities are not necessarily mutually exclusive as the processing of both internal and external information could be complimentary (Mantini and Vanduffel, 2013). Consistent with our findings, similar integration between the default-mode network and task-positive regions was also observed in Bluhm et al. (2011) in a working memory task, Fornito et al. (2012) in a recollection task, Spreng et al. (2010)in a autobiographic planning task, and Gao and Lin (2012) in a movie watching task. Taken together, the default-mode network seems to participate in different domains of functions through active coupling with outside network regions although the exact role of this coupling during different tasks remains to be determined and differentiated.

### The relationship between task-dependent default-mode network reorganization and behavior

Our results indicate significant negative correlation between the degree of within-network desynchronization and RT as well as significant positive correlation between outside-network integration and accuracy (Figure 7). These brain-behavior relationships strongly support the behavioral importance of the dynamic task-dependent reorganization pattern of the default-mode network observed in this study. More importantly, we have identified interesting disassociated behavioral effect (Figure 7); connection changes representing within-network desynchronizations only correlate with RT while connection changes representing outside-network integrations only correlate with accuracy. The former pattern indicates that the more disrupted the connections between stable regions and “decreasing regions”, the faster (shorter RT) the subjects perform the task. This is plausible since the suppression of task-irrelevant self-related thinking processes supported by the “decreasing” regions likely leads to more resources for external stimuli processing hence improved reaction time. On the other hand, stronger integration between stable default-mode network regions and outside-network task-positive regions is associated with increased accuracy. This pattern also makes sense if we consider that these areas are important for cognitive control functions that are essential for correct classification of letters (e.g., conflict monitoring, error perception/detection, as described above). In terms of the default-mode network, this particular behavioral correlation may bias our interpretation of its active role toward help in monitoring external environment although the alternative possibility (i.e., assessment of internal stimuli) is possibly complementary (Mantini and Vanduffel, 2013) and also deserves attention.

### Limitations

The highly convergent findings from both seed-based analysis and data-driven ICA analysis (Figures 2, 3) strongly support the robustness of the reported dynamic patterns related to the default-mode network. However, one potential confounding factor in the interpretation of the task-dependent changes in correlation values as observed in this study is the potential change in the signal-to-noise ratio (SNR) during different brain states: increasing/decreasing SNR in a given region may increase/decrease the correlation magnitude between this region and other areas. However, in our results, for the same set of stable regions during the same brain state shift, both significant increase and decrease in correlation magnitude were observed (Figures 4, 5). Therefore, potential task-related SNR change in the stable regions alone could not explain the observed regional specific and bi-directional changes in correlations. However, future studies with well-defined trial structures may be needed to quantitatively evaluate the effect of SNR change on the dynamic changes of correlations.

Moreover, the current study focused only on the default-mode network. However, the fact that the “increasing” regions generally reside in a previously reported “salience” network (Seeley et al., 2007) is intriguing. Such findings may imply that the default-mode network's dynamic reorganization, especially regarding its increased coupling with other parts of the brain, may behave at the network level. Therefore, more dedicated network-level analyses including multiple related systems such as the salience network (Seeley et al., 2007), the executive control network (Seeley et al., 2007), and the dorsal attention network (Fox et al., 2005), as previously done by us and others (Spreng et al., 2010; Gao and Lin, 2012) is highly deserved and will be one of our future directions. Finally, regarding the controversy of global signal regression (Chang and Glover, 2009; Fox et al., 2009; Murphy et al., 2009), we repeated our analyses without this step. It turned out that although the absolute functional connectivity values shifted toward being more positive, the resulting task-dynamic changes in functional connectivity across different tasks remain highly consistent with the reported results, which is also in line with our previous findings regarding this matter based on a very similar experimental design (Gao and Lin, 2012). Finally, the potential outside-network integration pattern of the default-mode network during different external attention-demanding tasks likely differ depending on the nature and functional recruitment of the task (Spreng et al., 2010; Bluhm et al., 2011; Fornito et al., 2012; Gao and Lin, 2012). For example, Sala-Llonch et al. (2012) reported that connections between the default network and working memory regions became increasingly negative during working memory tasks and the stronger the negative correlations the better the performance. Therefore such task-type-dependent dynamic network-level interaction patterns of the default network deserve further investigation.

## Conclusions

Overall, we have provided evidence for the dynamic reorganization of the default-mode network under different experimental states characterized by within-network desynchronization and outside-network integration. Such dynamic default-mode network reorganization patterns significantly and differentially correlate with task performance measures strongly supporting their behavioral significance. Our results suggest that versatile functional roles of the default-mode network are partially supported by its context-dependent dynamic changes of connectivity pattern either within or across network boundaries, which emphasizes the importance of a dynamic perspective in future search of the default-mode network's functioning mechanism.

### Conflict of interest statement

The authors declare that the research was conducted in the absence of any commercial or financial relationships that could be construed as a potential conflict of interest.
